# Oral health and oral health-related habits of Finnish prisoners

**DOI:** 10.1038/bdjopen.2017.6

**Published:** 2017-03-03

**Authors:** Raija Vainionpää, Arto Peltokangas, Jukka Leinonen, Paula Pesonen, Marja-Liisa Laitala, Vuokko Anttonen

**Affiliations:** 1 Research Unit of Oral Health Sciences, Department of Cariology, Endodontology and Paediatric Dentistry, University of Oulu, Oulu, Finland; 2 Research Unit of Oral Health Sciences, University of Oulu, Oulu, Finland; 3 Medical Research Centre, Oulu University Hospital and University of Oulu, Oulu, Finland

## Abstract

**Objectives/Aims:**

This cross-sectional study aimed to examine oral health and oral health-related habits among prisoners at the Pelso Prison in Finland.

**Materials and Methods:**

Participants in this cross-sectional study comprises 100 inmates. A calibrated dentist recorded the decayed, filled and missed teeth as well as periodontal status (bleeding-on-probing, pocket probing and Community Periodontal Index) among the participants (*n*=100). Fifty inmates were also interviewed for marital status, education and oral health-related habits. The Ethical Committee of the Northern Ostrobothnia Hospital District and the Criminal Sanctions Agency approved the study protocol.

**Results:**

The participants were on average 35 years old and had 5 (s.d. 5.1) decayed teeth in need of restorative treatment, whereas DMFT was 17 (s.d. 8.9). Half of the study population had periodontal disease in need of professional treatment. Almost all reported brushing their teeth daily. Two-third ate sweets, one-third drank fizzy drinks and majority smoked every day. Almost two-third had used illicit drugs at some point of their lives. Almost all drunk alcohol once a week or more often. No statistically significant associations were discovered between dental treatment need and explanatory factors.

**Discussion:**

Prisoners appear to be a homogenous group with poor oral health and harmful health behaviours.

## Introduction

At present, the oral health of Finnish adults has been reported to be good, although their dentitions are heavily restored (Health Survey 2011).^1^ However, little is known about the oral health of prisoners in Finland. At the end of 2014, there were 2,851 prisoners in Finland (Criminal Sanctions Agency, 2014).^2^ Males dominated the criminal populations and only 7.7% of the Finnish prisoners were women.^2^ The mean age of Finnish prisoners is ~37 years, women being on average 2 years older than men. Lower social classes are over-presented in the prison population.^3^ Almost all (94%) prisoners in Finland have a psychiatric diagnose, abuse of recreational drugs being most common followed by personality disorders.^4^

The literature on oral health of the prisoners in English published between 1995 and December 2015 in the MEDLINE and PubMed databases revealed 91 articles. Fourteen of them had similar study populations to the present study, as well as DMFT and MT values of the inmates; 11 articles also reported Community Periodontal Index (CPI) values. The keywords used in the searches were: prison, prisoner, inmate, caries, periodontal disease, oral-related habit, intoxicant, tooth brushing, diet, fizzy drink, sweet and snack. None of the 91 derived from the Nordic countries. The studies reported that the clinical oral health status and self-reported oral health of prisoners is poor and poorer than among age-matched non-prisoner individuals (Table 1).^3,5–17^ The mean DMFT varied from 2.3 to 22.5. The mean MT varied from 2.5 to 20.3. Periodontal health was studied using a variety of methods in different studies, but in general, the proportion of those with CPI⩾3 was 40%.

Our aim was to examine oral health and oral health-related behaviours among the prisoners at the Pelso Prison in Vaala, Finland. Our hypothesis was that the oral health status of prisoners at the Pelso Prison is poor and generally poorer than among age-matched Finnish adult population. Furthermore, we hypothesised that oral health-related habits are poor among prisoners.

## Materials and methods

### Subjects

The study population in this cross-sectional clinical study comprises 100 prisoners: 89 males and 11 females, who all were Finnish citizens—the study population can be considered a comfort sample, where all inmates imprisoned during the period September 2014 and February 2015 were invited to participate. Pelso prison is a closed prison with facilities for 110 prisoners including a department for 10 female prisoners. The participating prisoners were both long- and short-stay prisoners, but details of their sentences were not available for the research group. In total, 100 prisoners (all volunteering to participate) were clinically examined and 50 of them, according to the time limit, were also interviewed. Four subjects refused to participate in the clinical examination and one in the interview. The data collection was conducted during the period September 2014 and February 2015.

### Examiner training, calibration process and validation

One examiner (author RV, working as a dentist in Pelso prison) performed all clinical examinations. Before the examinations, an experienced specialist (author VA) trained the examiner (author RV) on the study protocol and diagnostic criteria using a PowerPoint presentation. Different stages of dental caries (ICDAS) were demonstrated using extracted teeth that had been cut into halves. Five volunteer prisoners were invited to test the protocol and demonstrate the clinical examination (authors RV, VA and MLL). Inter- and intra-examiner agreement (kappa values) was calculated for dental caries and periodontal status. For the inter-examiner agreement, authors RV and MLL examined a total of 10 patients in three occasions ~2 months apart, and for the intra-examiner agreement, author RV re-examined every 10th patient.

### Clinical examinations

The clinical examinations were carried out at the fully equipped dental office of the Pelso Prison. For the oral examination, the light of the dental unit, three-in-one-syringe, oral mirror, World Health Organisation gingival probe and fibre optic trans-illumination were used. Bitewing radiography was used when at least one lesion was clinically observed to extend into the dentin. The films were developed in an automatic processor, the Dürr Dental Periomat Plus. The radiographs were analysed by RV.

For recording caries findings, the ICDAS criteria and activity estimation were used for all dried tooth surfaces. Score 0 represented sound surface, enamel lesions comprises scores 1 and 2, and inactive score 3, lesions needing restorative treatment comprises active score 3 and 4–6 (D). Restorations and materials and missing teeth were recorded. Teeth were not cleaned professionally before the examination.

Periodontal health was examined as follows: for the bleeding-on-probing score (BOP), all tooth surfaces (buccal, lingual and interproximal surfaces) were probed by a World Health Organisation gingival probe using weight of ~20 g. The weight was calibrated using a digital letter scale during the training session and occasionally during the clinical examinations. Gingival bleeding was evaluated after probing all teeth by an oral mirror. The number of bleeding sextants was registered. Findings of each sextant were examined if there were two or more teeth present. Here BOP values instead of CPI values have been used, because BOP has been shown to predict future periodontitis in follow-up studies specifically among young adults.^18^ Calculus and the depth of pockets were measured and registered as a CPI advised by Ainamo *et al.*^19^ and the World Health Organisation.^20^ CPI was recorded using values from 0 to 4 or the letter X (=less than two teeth present). 0=Healthy; 1=Bleeding observed; 2=Calculus detected during probing with pocket depth <4 mm; 3=Probing depth of 4–5 mm in at least one tooth/sextant; and 4=Probing depth of 6 mm or more in at least one tooth/sextant. If CPI value exceeded 4, a full periodontal status was registered (gingival pockets and mobility).

### Survey

Oral health-related habits and health behaviours were investigated by using a structured formula. Interviewing the participants instead of letting them answer a questionnaire was necessary because some of the inmates could not even read or write properly. Age (years), marital status (married or co-habiting/single or widowed or divorced), education (compulsory or vocational school/matriculation exam or gymnasium) and oral-related habits such as teeth brushing, consumption of snacks (sweets and fizzy drinks) and use of intoxicants (smoking/snuffing/alcohol/drugs) were surveyed using a one-on-one oral interview with the author RV.

Frequency of tooth brushing was surveyed using the following questions: How many times per day do you brush your teeth: once a day/twice a day/more than twice a day? When do you brush your teeth: in the morning/in the daytime/in the evening? How often do you brush your teeth: never or hardly ever/every day or almost every day/occasionally during the week? The alternatives for the question concerning the consumption of fizzy drinks and sweets were as follows: never or nearly never/every day or almost every day/occasionally during the week. Use of intoxicants (smoking, snus, drugs and alcohol) was surveyed with the following questions: Do you smoke: no/1–5 cigarettes daily/10–20 cigarettes daily/more than 20 cigarettes daily? Do you use snus: yes/no? Do you use drugs: yes/no? Do you use alcohol: no/twice a month or less frequently/once a week or more frequently?

### Ethical approval

The participation was voluntary, and all prisoners at the Pelso Prison were given the opportunity to participate. All the participants gave their informed consent. The Criminal Sanctions Agency and Ethical Committee of the Northern Ostrobothnia Hospital District gave their approval to the study (respectively, on 8 August 2014 and 16 June 2014, EETTMK: 50/2014).

### Statistical methods

In the literature review (Table 1), if CPI values had not been given, CPI values 1 and 2 were interpreted to represent mild gingivitis, CPI=3 moderate and CPI=4 severe periodontitis. BOP used here was not present in literature. For the validity of the clinical examinations, the intra-examiner and inter-examiner agreements were investigated by calculating kappa values (*κ*). Decayed, missing and filled teeth, i.e., DMFT indices were calculated tooth-wise as follows: ICDAS 0–3→D=0 (sound); 4–6→D=1 (caries lesion needing restoration); missing tooth and restored tooth without caries→M=1 and F=1, respectively. The DT and DMFT were described with means (s.d.), 95% CI, minimum and maximum values. The frequencies and distributions in terms of BOP and CPI values, oral health-related habits, and socioeconomic variables were reported. The outcome measures were restorative and periodontal treatment needs (ICDAS⩾4 and CPI⩾2, respectively). The association between the outcome and explanatory variables was analysed using cross tabulation with Pearson *χ*^2^ and Fisher’s exact tests. The association between the variables was considered statistically significant at *P* levels <0.05. All the statistical analyses were performed using the SPSS (version 22.0, SPSS, Chicago, IL, USA).

## Results

Our study population was dominated by male prisoners (89.0%). The mean age of the males was 35 years old (minimum 21 and maximum 70) and of the women 38 years old (minimum 23, maximum 61). A third of the inmates (33.0%) were younger than 30 years old.

Nearly two-third of the respondents (64%) were single. The single status was more common among the older prisoners (⩾30 years). Only two (male) inmates (4%) had done the matriculation exam, 28% had vocational school education and two-third (68%) had completed the compulsory school education of 9 years. The educational status of the female prisoners was even lower as all of them had only completed the compulsory school education (Table 2).

All but one of the respondents (98.0%) reported brushing their teeth every day but less than half (40.0%) twice a day. Nearly two-third (60.0%) of the interviewed ate sweets every day. More than two-third (72.0%) reported drinking fizzy drinks never or hardly ever. Majority of the respondents (88.0%) smoked and a fifth (20.0%) reported using snuff or smokeless tobacco. Nearly two-third (62%) had used illicit drugs over the course of their life. Nearly half of the respondents (40.8%) reported drinking alcohol prior to imprisonment once in a week or more often.

### Caries and periodontal status

*κ* value for the intra-examiner agreement was 0.79 for ICDAS and 0.48 for BOP. For inter-examiner agreement, *κ* value was 0.82 for ICDAS and 0.55 for BOP. Table 2 shows the distribution of the mean DT, DMFT, CPI and BOP values, and oral health-related behaviours among the study population. The proportion of prisoners with no need for restorative treatment was 19%. None of the inmates were edentate. The mean DT was higher for the males than for the females. DT increased with age among the female prisoners, but this was not seen among the male prisoners. More than half of the study population (57.0%) had three or more decayed teeth. The mean DT was 5.0 (s.d. 5.11), FT 6.9 (s.d. 5.15) and MT 4.7 (s.d. 6.01). Almost all (93.0%) of the study population had CPI score of 2 or more representing a need for invasive periodontal treatment. Respectively almost 94% had bleeding in four or more sextants.

## Discussion

At present, the literature on the oral health status of Finnish prisoners is non-existent which adds value to this study revealing that inmates appear as a homogenous group with both poor oral health and somewhat harmful oral health-related behaviours. Another strength of the study is that 100 of the 110 inmates participated in the study and 50 of the 100 clinically examined inmates were also interviewed using a structured formula for their oral health-related habits and health behaviours. Studies from other countries report that oral health status of prisoners is worse than that of non-prisoners, which is in concordance with our findings as well as our hypothesis.

In the Health Survey 2011^1^ in Finland, the mean DT value for men aged 30–34 years was 1.0 and for women 0.3, which are remarkably lower compared with the figures among the prisoners (5.1 for the males below 30 and 2.3 for the females). This study demonstrates remarkable need for restorative treatment among Finnish prisoners. Surprisingly, the mean DT value among men decreased slightly with age, but increased significantly among women. Viitanen *et al.*^21^ reported that general health among Finnish female prisoners is poor too, which turned out to be the case as for their oral health, according to this study. However, due to the limited number of the female participants, generalisations cannot be made. According to previous studies, there are differences in the oral health of short- and long-stay prisoners.^10^ In the Pelso prison, both types are present; unfortunately, details of this were not available for the research group, which is a shortcoming and makes impossible to investigate association of sentence type with oral health. This could be a topic for a future study.

One author (RV) performed all examinations, which could be considered a source of bias. However, quality assurance showed both good reliability and fair to good reproducibility. As for BOP, time difference between examinations may have caused actual changes in BOP. The mean DMFT index value of Pelso prisoners was high (16.8), but similar to that of prisoners in China (22.5),^3^ the United States (16.8),^5^ Great Britain (12.2 and 14.2),^8,14^ France (12.8),^13^ Brazil (20.4 and 19.7),^16,17^ Australia (20.4),^6^ Italy (9.8)^9^ and South Africa (15.5).^7^ On the other hand, lower DMFT values were found in studies in Nigeria (2.3)^15^ and India (3.36 and 5.26).^11,12^ The differences in these results can partly be explained by cultural and geographical differences. Study designs may also have caused differences. However, Kassebaum *et al.*^22^ demonstrated huge differences in caries status around the world in primary and permanent dentitions. The prevalence of periodontitis at the population level has been reported to be high among adult Finns: almost half of the Finnish women (43.0%) and two-third of the Finnish men (57.0%) have periodontitis.^1^ In this respect, prisoners do not differ from adult Finns.

According to the survey, the second hypothesis (oral health-related habits are poor among prisoners) appears to be true. Oral hygiene habits have improved among adults in Finland over the last decade: more than half of males and four-fifth of females report brushing their teeth twice daily,^1^ when in this study the proportion is less than half. Therefore, in addition to invasive professional restorative and periodontal treatment, most prisoners would benefit from personal oral hygiene instruction. Detailed periodontal status will be a topic for a future survey.

Unhealthy diets seem to be common among prisoners according to our findings; for example, two out of three reported eating sweets every day or almost every day. An alarmingly high proportion of the study population (88%) smoked. This is distinctly more than that among the adult population in the Health Survey 2011,^1^ where 17% of Finnish men and 14% of women aged 30 or more reported smoking every day. Prevalence of smoking has decreased clearly between 2000 and 2011 among Finnish men in all age groups,^1^ which does not seem to include this marginal group. On the other hand, it seems that use of snuff among the young Finnish men is increasing.^23^ In this study, 20% of the respondents reported using snuff. The effect of smoking on oral health, and particularly on periodontal health, is widely accepted.^23,24^ This was also demonstrated among Nigerian prisoners.^15^ Smoking is also associated with other harmful health behaviours and low educational status.^22^

Nearly half of the inmates reported drinking alcohol once a week or more often. High frequency of alcohol consumption is associated with caries, periodontal disease, tooth wear and xerostomia as well as traumatised teeth.^25^ Abuse of intoxicants among Finnish prisoners is 10 times more prevalent in comparison with the general population.^4^ Two-third of the interviewed prisoners had used illicit drugs at some point of their lives. The use of illicit drugs leads to poor oral hygiene and diet, which in turn leads to caries and periodontal disease. Drugs disguise dental pain, which explains in part why drug users visit the dentist rarely and irregularly.^25^

More than half of the study population were single. There were more single people among prisoners over 30 than among the younger ones. In the 31-year survey conducted on the North Finland 1966 birth cohort, the single marital status was associated with poor self-reported oral health, which is in concordance with our findings, even if statistical significance was not discovered.^26^ This study also supports earlier reports on fairly low socioeconomic status among inmates.^3^ In Finland, socioeconomic status is known to associate with educational level and in this study population, only two (out of 50 or 4%) had completed matriculation examination, when the proportion among general population in 2007 was 28.3% among those 15 years or older and can be even 50% among younger age cohorts (National Finnish Statistics; http://www.stat.fi/til/lop/2008/lop_2008_2009-06-12_tau_004.html). In health promotion, it must be kept in mind that the target group may be illiterate.

## Conclusions

This study shows that prisoners in Finland can be considered a homogenous group in terms of the prevalence of dental caries and periodontal disease as well as harmful health behaviours and poor oral hygiene. Four in five have the need for restorative treatment and not one had healthy periodontium. Health behaviours and educational status of Finnish prisoners are considerably poorer than those of the general population. Prison health services should and could improve the oral health of prisoners,^27^ yet the resources are often limited and are mainly used for emergency care. However, one must remember that a prisoner is also first and foremost a patient in need of clinical treatment.

## Figures and Tables

**Table 1:** Literature review

*References*	*Year*	*Country*	n	*Age, years (s.d.)*	*DMFT,* n *(s.d.)*	*MT (s.d.)*	*CPI*
McGrath^3^	2002	China	64	⩾60	23 (10.6)	20 (1.4)	40% CPI 3–4
Heng *et al.*^5^	2002	United States	F, 500	36	17 (7.3)	7 (7.0)	
Osborn *et al.*^6^	2003	Australia	M, 657 F, 132	M, 34 (12.4) F, 33 (10.5)	20	9	Mean CPI 2.2
Naidoo *et al.*^7^	2005	South Africa	M, 264 F, 76	15–35	16	—	8% CPI=0 56% CPI=1.2 31% CPI=3 3% CPI=4
Jones *et al.*^8^	2005	UK	326		M, 15 F, 18	9	
Nobile *et al.*^9^	2007	Italy	M, 544	39 (10.7)	10 (6.1)	6.0 (5.9)	10.5% CPI=0 2.5% CPI⩾4
Heidari *et al.*^10^	2007	GB	78	36 (9.6)	14 (7.5)	6.2 (7.6)	39.0% CPI=3 2.5% CPI=4
Bansal *et al.*^11^	2012	India	M, 1278 F, 115	35 (12.3)	35–44 years: 3 (3.3) 65–74 years: 4 (3.9)	1 (2.1)	
Reddy *et al.*^12^	2012	India	M, 722 F, 78		5 (2.9)	57.1% ⩾1	39.3% CPI=2 46.9% CPI 3–4
Decerle *et al.*^13^	2012	France	M, 84	39 (13)	12.8 (7.8)	6.3 (7.1)	49% CPI 3–4
Rouxel *et al.*^14^	2013	UK	F, 103	30.9 (9.6)	12.3 (7.5)	4.96 (4.92)	62% CPI=3 15% CPI=4
Akaji *et al.*^15^	2013	Nigeria	230 S, 120 NS, 90	28.5 (9.5)	2.31 (0.77) S, 2.4 (0.71) NS, 2.3 (0.83)		CPI 3.5 (1.1) S, 4.7 (1.3) NS, 2.3 (0.9)
Rodrigues *et al.*^16^	2014	Brazil	F, 65	32.2 (11.6)	20.4 (7.9)	11.3 (10.4)	
Cavalcanti *et al.*^17^	2014	Brazil	M, 127	28.5 (7.8)	19.7 (6.3)	7.2 (7.2)	

Abbreviations: CPI, Community Periodontal Index; F, Female; M, male; Non-S, non-smoker; S, smoker.

**Table 2:** Distribution of participants according to age, gender, oral health and background factors

*Variable*	*<30 years*			*⩾30 years*			*Total (*n*=100)*
	*Male (*n*=31)*	*Female (*n*=3)*	*Total (*n*=34)*	*Male (*n*=58)*	*Female (*n*=8)*	*Total (*n*=66)*	
*DT*							
Mean (s.d.)	5.1 (5.97)	2.3 (2.52)	4.8 (5.76)	4.8 (4.44)	7.4 (7.04)	5.1 (4.80)	5.0 (5.11)
Minimum, maximum	0, 25	0, 5	0, 25	0, 17	0, 19	0, 19	0, 25
							
*DMFT*							
Mean (s.d.)	12.3 (7.80)	13.7 (3.79)	12.4 (7.50)	18.7 (8.87)	21.1 (8.44)	19.0 (8.80)	16.8 (8.90)
Minimum, maximum	1, 31	11, 8	1, 31	1, 32	9, 30	1, 32	1, 32
							
*CPI*							
Max 1: *n* (%)	1 (3.2)	0	1 (2.9)	6 (10.3)	0	6 (9.1)	7 (7.0)
Max 2: *n* (%)	21 (67.4)	2 (66.7)	23 (67.7)	27 (46.6)	2 (25.0)	29 (49.9)	52 (52.0)
Max 3: *n* (%)	8 (25.8)	1 (33.3)	9 (26.5)	20 (34.5)	5 (62.5)	25 (25.3)	34 (34.0)
Max 4: *n* (%)	1 (3.2)	0	1 (2.9)	5 (8.6)	1 (12.5)	6 (9.1)	7 (7.0)
							
*BOP*							
0–1 Sextants: *n* (%)	1 (3.3)	0	1 (3.0)	5 (8.5)	0	5 (7.5)	6 (6.0)
2–3 Sextants: *n* (%)	1 (3.3)	0	1 (3.0)	3 (5.1)	2 (25.0)	5 (7.5)	6 (6.0)
4–6 Sextants: *n* (%)	28 (93.3)	3 (100.0)	31 (93.9)	51 (86.4)	6 (75.0)	57 (85.1)	88 (88.0)
	*n*=17	*n*=1	*n*=18	*n*=29	*n*=3	*n*=32	*n*=50
							
*Sweets*							
Never/hardly ever	3 (17.6)	1 (100.0)	4 (22.2)	14 (48.3)	2 (66.7)	16 (24.2)	20 (40.0)
Occasionally during week	9 (53.0)	0	9 (50.0)	12 (41.4)	1 (33.3)	13 (19.7)	22 (44.0)
Every or almost every day	5 (29.4)	0	5 (27.8)	3 (10.3)	0	3 (4.6)	8 (16.0)
							
*Fizzy drinks*							
Never/hardly ever	14 (82.4)	1 (100.0)	15 (83.3)	19 (65.5)	2 (66.7)	21 (65.6)	36 (72.0)
Occasionally during week	3 (17.6)	0	3 (16.7)	7 (24.1)	1 (33.3)	8 (25.0)	11 (22.0)
Every or almost every day	0	0	0	3 (10.3)	0	3 (9.4)	3 (6.0)
							
*Tooth brushing*							
Never/hardly ever	0	0	0	0	0	0	0
Occasionally during week	0	0	0	1	0	1	1 (2.0)
Every or almost every day	17	1	18	28	3	31	49 (98.0)
							
*Tooth brushing*							
Morning	11	1	12	15	2	17	29 (58.0)
Daytime	1	0	1	1	0	1	2 (4.0)
Evening	15	1	16	24	3	27	43 (86.0)
							
*Marital status*							
Married or co-habiting: *n* (%)	7 (41.2)	1 (100.0)	7 (38.9)	7 (24.1)	3 (100.0)	10 (31.3)	17 (34.0)
Single: Widow/er or divorced: *n* (%)	10 (58.8)	0	11 (61.0)	22 (75.9)	0	22 (68.7)	33 (66.0)
							
*Education*							
Compulsory vocational	17 (100.0)	1 (100.0)	18 (100.0)	27 (93.1)	3 (100.0)	30 (93.7)	48 (96.0)
Matriculation exam or gymnasium	0	0	0	2 (6.9)	0	2 (6.3)	2 (4.0)

Abbreviations: BOP, bleeding-on-probing; CPI, Community Periodontal Index.
